# Synthesis and characterization of some novel fatty acid analogues: A preliminary investigation on their activity against human lung carcinoma cell line

**DOI:** 10.1186/1476-511X-12-45

**Published:** 2013-03-28

**Authors:** Selvaraj Jubie, Palanisamy Dhanabal, Mohammed Afzal Azam, Nithyanantham Muruganantham, Rajagopal Kalirajan, Kannan Elango

**Affiliations:** 1 Department of Pharmaceutical Chemistry, JSS College of Pharmacy, Udhagamandalam, Rock lands, Ooty 643 001, Tamilnadu, India; 2 Department of Phytopharmacy and Phytomedicine, JSS College of Pharmacy, Udhagamandalam, Tamilnadu, India

**Keywords:** *Spirulina platensis*, Stearic acid, Gamma linolenic acid, Cytotoxicity

## Abstract

**Background:**

Preparation of some novel heterocyclic compounds with long alkyl and alkenyl chain of cytotoxic activity.

**Methods:**

Gamma linolenic acid, a poly unsaturated fatty acid and stearic acid, a saturated fatty acid were isolated from the microalga *Spirulina platensis*. Some novel gamma linolenic acid and stearic acid analogues having 1,3,4-oxadiazole and 1,2,4-triazole were synthesized and characterized by IR, ^1^H NMR, ^13^C NMR and mass spectral analysis. Cytotoxicity of these compounds was evaluated by the growth inhibition of A-549 cells *in-vitro*.

**Results:**

Compound **1** and **3** showed comparable cytotoxicity against the human lung carcinoma A-549 cell lines.

## Background

*Spirulina platensis*, blue green microalgae is being widely studied, not only for nutritional reasons but also for its reported medicinal properties [1]. It is a potential source of GLA (Gamma linolenic acid), an essential polyunsaturated fatty acid of excellent economic interest [2]. *In-vitro* and *in-vivo* studies have shown GLA to selectively kill tumor cells without harming normal cells [3]. Natural sources of GLA contain variable amounts of this acid which rarely exceed 25%; hence there has been a keen interest in producing higher concentrates of GLA. The commercial methods for producing GLA concentrates include winterization, fractional distillation, urea-inclusion, high performance liquid chromatography and argentated silica gel chromatography [4]. GLA concentrations mentioned above are sufficient for most applications; however several uses, particularly pharmaceutical applications require higher concentrations of GLA, often in excess of 90% [5]. Recently Centrifugal chromatography system was shown to play a vital role in extracting phyto constituents from natural sources.

Although billions of dollars have been spent on research and development on anticancer drugs, the disease remains uncontrolled. A number of investigations have demonstrated that, a variety of modified fatty acid analogues are promising molecules in cancer prevention and have potential in the treatment of cancer [6,7]. During the last two decades, the chemistry of 1,2,4-triazole, 1,3,4-oxadiazole and their derivatives have received considerable attention owing to their anticancer activities [8-10].

Based on these findings and continuation of our previous work [11], the present work has been aimed to develop some novel 1,2,4-triazoles and 1,3,4 oxadiazoles synthesized from GLA which is isolated from *Spirulina platensis*, and evaluate their anticancer activity. GLA was isolated from *Spirulina platensis* by a novel method using Cyclograph centrifugal chromatography system.

## Results and Discussion

### Isolation of GLA methyl ester

Fatty acid methyl esters (FAME) were prepared from freeze dried biomass of *Spirulina platensis*, which was then subjected to urea fractionation for getting gamma linolenic acid methyl ester by extraction with n-hexane. The n-hexane fraction was quantified by HPTLC and was found to contain 57.62% w/w of GLA methyl ester. The GLA methyl ester was separated by Cyclograph Centrifugal Chromatography System, which combines the advantages of both preparative TLC and column chromatography. It delivers fast and efficient separations. Centrifugal action combined with the use of a solvent pump to apply the mobile phase allows complete control of solvent velocity profile.

### Chemistry

**Scheme 1:**
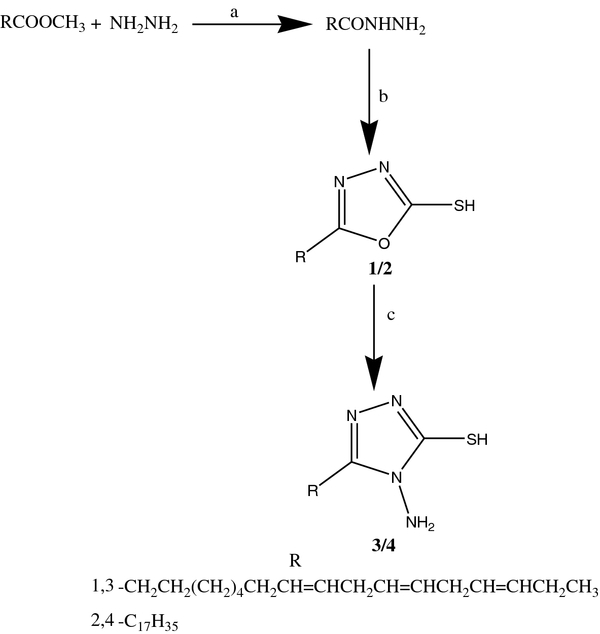
**Synthesis of the compounds.** Reagents and conditions. **a)** NH_2_NH_2_, reflux 8 h. **b)** POCl_3_, C_17_H_35_COOH, reflux, 8 h, neutralization with NaOH, **c)** NH_2_NH_2_, reflux, 8 h, acidification with HCl upto pH3.

Four novel fatty acid analogues have been synthesized such as 5 (−heptadeca-5,8,11-trienyl)-1,3,4-oxadiazole-2-thiol, 5 (heptadecyl)-1,3,4-oxadiazole-2-thiol, 5-heptadeca-5,8,11-trienyl)-4-amino-1,2,4-triazole-3-thiol and 5-(heptadecyl)-4-amino-1,2,4-triazole-3-thiol as illustrated in Scheme 1. Acid hydrazides, the key intermediates for our synthesis were prepared and used for subsequent cyclization reaction to yield the corresponding 1,3,4-oxadiazoles without separation. Intramolecular cyclization of acid hydrazides with carbon disulphide resulted in the corresponding oxadiazoles **(1&3)**. Compounds **1&2** were treated with two-fold excess hydrazine hydrate in ethanol to yield the triazoles **(2&4)**.

### *In-vitro* cytotoxicity screening

Compounds **1, 2, 3** and **4**, were tested for cytotoxic potential in A549 (lung adenocarcinoma) cells by determination of CTC_50_ (concentration of the sample required to kill 50% of the cells) by SRB assay. The experiments were carried out in triplicate and the results are presented in Table 1. The compounds showed dose-dependent destruction of the cell. The 1,3,4-oxadiazole substituted fatty acid analogues **1** and **3** showed maximum cytotoxic activity. The presence of toxophoric –N=C-O- linkage in 1,3,4-oxadiazole nucleus may be responsible for the activity. Further, 1,3,4-oxadiazole is a good bioisostere of amide and ester functionalities with substantial improvement in biological activity in hydrogen bonding interactions with different receptors. It is also observed that the length of the fatty acids play a vital role in anti-tumor activity. The 1,2,4-triazole substituted fatty acid analogues **2 **and **4** displayed promising cytotoxicity.

**Table 1:** Determination of cytotoxicity by SRB method

**Compound**	**CTC**_**50 **_**(μM)**
1	0.149
2	0.264
3	0.143
4	0.282

CTC_50_-concentration of the sample required to kill 50% of the cell.

## Conclusion

In conclusion, 5-heptadeca-5,8,11-trienyl/heptadecyl)-1,3,4-oxadiazole-2-thiol **1**–**2 and** 5-(heptadecyl/-heptadeca-5, 8,11-trienyl)-4-amino-1,2,4-triazole-3-thiol **3**–**4** were synthesized from the methyl esters of gamma linolenic acid and stearic acid which were isolated from the microalga *Spirulina platensis*. The compounds were evaluated for their *in-vitro* cytotoxicity by SRB method on human lung carcinoma cell lines (A-549). The 1,3,4-oxadiazole substituted compounds **1** and **3** showed potent cytotoxicity.

## Materials and methods

### Materials

All chemicals used were purchased from Fluka chemicals. Their purity was checked by GC. All solvents were purified by distillation and if necessary residual water was removed. The composition of solvents and eluents are given in volume ratios of the components. Fresh cultures of *Spirulina platensis* was obtained from Antenna Research Foundation Pvt Ltd., Madurai, Tamilnadu, India.The cell paste was lyophilized and stored at −20°C for further use. Stearic acid ester was isolated by previously reported method [11]. GLA ester was isolated by using Cyclograph Centrifugal Chromatography System (Analtech inc). Products were purified by the column chromatography and identified using different spectral techniques.

Melting points were taken in glass capillary tubes on a Veego VMP-1 apparatus and are uncorrected. The ^1^H NMR and ^13^C NMR were recorded on Bruker DRX-300 (300 MHZ FT-NMR) using deuterated chloroform as solvent and TMS as internal standard. The mass spectra of compounds were recorded on JEOL GC MATE II GC-MS.

### General procedure for isolation of GLA methyl ester

**Figure 1:**
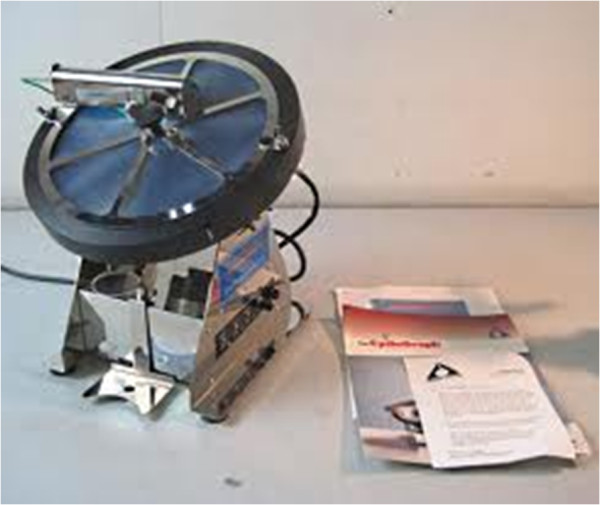
Cyclograph centrifugal chromatography.

The cyclograph system is a centrifugally accelerated device for performing preparative chromatographic separations. The device spins a layer of adsorbent material coated as a flat ring on a glass backing. A solvent pump is used to apply the sample and mobile phase to the centre of the spinning adsorbent ring. The centrifugal action accelerates the flow of the mobile phase through the adsorbent, separating the sample components as circular bands. The mobile phase elutes continuously into a specially shaped collection channel inside the body of the instrument. Component bands are collected manually in test tubes or optionally by an automated fraction collector (Figure 1).

### Preparation of fatty acid methyl esters (FAME)

Freeze dried biomass of *Spirulina platensis* (50 g) was extracted by reflux for 4 hours using a mixture of methanol and acetyl chloride (95:5, 800 ml). The extract obtained was diluted with water and extracted thrice with equal volume of n-hexane containing 0.01% butylated hydroxyl toluene. The combined n-hexane layer was evaporated to get the fatty acid methyl ester (FAME).

### Enrichment of FAME for GLA methyl ester

The FAME obtained was subjected to urea complexation as described previously to remove the saturated fatty acids from the polyunsaturated fatty acids. For the urea complexation, methanol (9 ml) and urea (3 g) were added to FAME. The mixture was heated to get a clear solution. It was cooled at room temperature and stored at 0°C overnight. Then it was filtered to remove the crystals settled at bottom. The filtrate was extracted with n-hexane containing 0.01% butylated hydroxyl toluene. The n-hexane fraction was evaluated for the presence of GLA methyl ester by HPTLC.

### Separation of GLA methyl ester

Enriched GLA fraction obtained above was directly applied in circular TLC plate placed in a cyclograph. The circular plate rotated using the motor and a constant flow rate of the mobile phase was used. Hexane was used as the mobile phase. Twenty fractions of volume 10 ml each were collected and evaluated for the presence of GLA methyl ester by HPTLC. GLA methyl ester was found to be present in fractions 12 to 17. Hence, these fractions were combined, hexane was evaporated and GLA methyl ester was obtained.

Viscous pale-yellow oil; isolated yield, 71%. ^1^H NMR (300 MHZ,CDCl_3_ δ_H_) ; 5.5 (m, 6H, olefinic), 3.7 (s, 3H, CH_3_ ester), 1.3 (m, 2H, CH_2_ next to CH_3_), 2.1 (m, 4H, CH_2_ adjacent to double bond), 0.9 (t, 3H, CH_3_ terminal), 2.3 (t, 2H, CH_2_ adjacent to terminal), 2.6 (t, 4H, CH_2_ between double bonds), 1.7 (m, 8H, C_3_, C_4_, C_16_ and C_17_) ppm. ^13^C NMR (300 MHZ, CDCl_3_ δ_C_): 22.53, 24.55, 24.75, 25.60, 26.85, 27.18, 29.21, 29.23, 29.23, 31.72, 51.51, 174.82, 130.41, 129.15, 127.56, 174.82, 174.82. FAB-MS: 289 (M-3, 100%): 254.70 [^+^(CH_2_)_4_CH=CHCH_2_CH=CHC(CH_2_)_4_COOCH_3_] *m/z*+1, 267(C_17_H_35_CN^+^ CH_3_(CH_2_)_4_CH=CHCH_2_CH=CHCH_2_CH=CH^+^) *m/z*+1,151.69 (C_6_H_5_CN^+^) *m/z*+1.

### General procedure for the synthesis of 5-heptadeca-5,8,11-trienyl/heptadecyl)-1,3,4-oxadiazole-2-thiol ***1****–****2***

Hydrazine hydrate (99%, 5 mmol) was placed in a round bottom flask fitted with a reflux condenser. To this solution methyl stearste/methyl gamma linolenate (5 mmol) was added dropwise and heated gently for 15 min. Then absolute ethanol (5 ml) was added through the condenser to produce clear solution and refluxed for 4–5 h. The ethanol was distilled off and cooled. To this stearic acid hydrazide/gamma linolenic acid hydrazide solution, a mixture of potassium hydroxide (5 mmol), carbon disulphide (5.51 mmol) and ethanol (20 ml) was added and further refluxed for 10 h. After concentration of the solution to small volume, the residue was dissolved in 10 ml of water. The precipitate was obtained by adding the solution to ice containing conc.HCl.

#### *5* [*heptadeca-5, 8, 11-trieny*]*-1, 3, 4-oxadiazole-2-thiol****1***

Viscous pale-yellow oil; R_f_ =0.46 (n-hexane/ethyl acetate, 1:1 v/v as developor), isolated yield, 71%. ^1^H NMR ( 300 MHZ, CDCl_3_ δ_H_) ; 0.9 (t, 3H, terminal CH_3_), 1.3 (t, 2H, CH_2_ next to ring), 1.3 (m, 8H, CH_2_ on C_3_, C_4_, C_16_ and C_17_), 1.4 (m, 2H, CH_2_ adjacent to terminal CH_3_), 2.1 (m, 4H, CH_2_ adjacent to terminal), 2.7 (m, 6H, CH_2_ adjacent to terminal), 2.8 (t, 4H, CH_2_ between double bonds), 1.3 (m, 8H, C_3_, C_4_, C_16_ and C_17_) ppm. ^13^C NMR (300 MHZ,CDCl_3_ δ_C_); 14.1, 22.6, 25.7, 28.6, 29.0, 29.7, 31.8, 32.4, 80, 127.4, 130. FAB-MS: 334.21 (M^+^, 100%): 248.33(^+^CH_3_(CH_2_)_4_CH=CHCH_2_CH=CHCH_2_CH=CH)CH_2_)_4_CH_2_+) *m/z*+1, 270.35(CH_3_(CH_2_)_4_CH=CHCH_2_CH=CH(CH_2_)_4_CH_2_^+^N=N) *m/z*-3, 231.38 (CH_3_(CH_2_)_4_CH=CHCH_2_CH=CHCH_2_CH=CH(CH_2_) *m/z*-2.

#### 5(heptadecyl)-1,3,4-oxadiazole-2-thiol **2**

Off-white solid; R_f_ =0.23 (n-hexane/ethyl acetate, 1:1 v/v as developor), isolated yield, 52%. m.p.76°C. ^1^H NMR (300 MHZ, CDCl_3_ δ_H_) ; 0.8 (t, 3H,terminal CH_3_), 1.2 (brm, 28H, CH_2_), 1.6 (m, 2H, CH_2_), 2.4 (t, 2H, CH_2_), 7.3 (s, 1H, SH) ppm. ^13^C NMR (300 MHZ, CDCl_3_ δ_C_); 14.09, 22.68, 25.69, 28.65, 29.02, 31.91, 76.39. FAB-MS: 340 (M^+^, 100%): 241 (C_17_H_35_) *m/z*+2, 251 (C_17_H_35_ C^+^) *m/z*.

### General procedure for the synthesis of 5-(heptadecyl/ (5Z,8Z,11Z)-heptadeca-5, 8,11-trienyl)-4-amino-1,2.4-triazole-3-thiol 3*–*4

To a mixture of appropriate oxadiazole **1/3** (10 mmol) in absolute ethanol (10 ml), hydrazine hydrate (10 mmol) was added and the reaction mixture was refluxed for 5 h. On completion of reaction potassium hydroxide (10 mmol) was added to the reaction mixture and the precipitate formed was filtered. The solid obtained was acidified with conc. HCl to pH-3 and washed with water. The resultant solid was recrystallised from ethanol.

#### 5 –(heptadeca-5, 8,11-trienyl)-4-amino-1,2.4-triazole-3-thiol **3**

Viscous pale-yellow oil; R_f_ =0.30 (n-hexane/ethyl acetate, 1:1 v/v as developor), isolated yield, 76%.^1^H NMR ( 300 MHZ, CDCl_3_ δ_H_) ; 0.9 (t, 3H, terminal CH_3_), 1.3 (m, 8H, CH_2_ on C_3_, C_4_, C_16_ and C_17_), 2.2 (s, 2H, NH_2_), 2.4 (m, 4H, CH_2_ adjacent to double bond), 2.6 (t, 4H, CH_2_ between double bonds), 4.1 (m, 2H, CH_2_ adjacent to terminal), 3.6 (t, 3H, CH_2_ next to ring) 5.3 (m, 6H, olefinic protons) 7.3 (s, 1H, SH) ppm. ^13^C NMR (300 MHZ, CDCl_3_ δ_C_); 14.1, 22.9, 23.0, 25.7, 27.8, 29.6, 29.7, 31.1, 32.0, 127.3, 128.8, 132.2. FAB-MS: 348.15 (M^+^, 100%): 248.33 [CH_3_(CH_2_)_4_CH=CHCH_2_CH=CHCH_2_CH=CH)CH_2_)_4_CH_2_^+^] *m/z*+1, 270.35 [CH_3_(CH_2_)_4_CH=CHCH_2_CH=CH(CH_2_)_4_CH_2_^+^N=N] *m/z*-3, 231.38 [CH_3_(CH_2_)_4_CH=CHCH_2_CH=CHCH_2_CH=CH(CH_2_] *m/z*-2.

#### 5--heptadecyl)-4-amino-1,2.4-triazole-3-thiol **4**

Off-white solid; R_f_ =0.21 (n-hexane/ethyl acetate, 1:1 v/v as developor), isolated yield, 72%. m.p.86°C. ^1^H NMR ( 300 MHZ, CDCl_3_ δ_H_) ; 0.9 (t, 3H,terminal CH_3_), 1.2 (brm, 28H, CH_2_), 1.6 (m, 2H, CH_2_), 2.4 (t, 2H, CH_2_), 5.2 (s, 1H, NH_2_), 7.3 (s, 1H, SH) ppm. ^13^C NMR (300 MHZ, CDCl_3_ δ_C_); 14.1, 22.8, 22.9, 29.3, 29.4, 29.7, 31.0, 31.9, 160.0, 167.4. FAB-MS: 354.38 (M^+^, 100%): 285.99 [C_17_H_35_CH_2_NHNH_2_] *m/z*+1, 264.96 [C_17_H_35_CN^+^] *m/z*-1,252.88 [C_17_H_35_CH^+^] *m/z*.

### Biology

#### Cell lines and culture medium

A-549 cell cultures used in the experiments were procured from National Centre for Cell Sciences, Pune, India. A-549 cells were grown in Earl’s Minimal Essential Medium supplemented with 2 mmol L-glutamine, 10% Fetal Bovine Serum, Penicillin (100 μg/mL), streptomycin (100 μg/mL) and amphotericin B (5 μg/mL) and the cells were maintained at 37°C in a humidified atmosphere with 5% CO_2_ and subculture twice a week.

#### *In-vitro* cytotoxicity screening

The total cell protein content was determined by Sulphoradamine B (SRB) assay [12,13]. The monolayer cell culture was trypsinized and the cell count adjusted to 1.0 × 10^5^ cell/mL using medium (MEM) containing 10% new born calf serum. To each well of the 96 well microtitre plate, 0.1ml of diluted cell suspension (approximately 10,000 cells) was added. After 24 h, when a partial monolayer was formed, the supernatant was flicked off, the monolayer was washed once and 100 μL of medium and the culture was exposed to different concentrations of drug in microtitre plates. The plates were then incubated at 37°C for 3 days in 5% CO_2_ atmosphere, and microscopic examination was carried out and observations recorded every 24 h. After 72 h, 25 μL of 50% trichloro acetic acid was added to the wells gently such that it forms a thin layer over the drug solution to give an overall concentration of 10%. The plates were incubated at 4°C for one hour. The culture plates were flicked and washed five times with tap water to remove traces of medium, drug and serum, and were then air-dried. The air-dried plates were stained with SRB for 30 min. The unbound dye was then removed by rapidly washing four times with 1% acetic acid. The plates were then air-dried. 100 μL of 10mM tris base was then added to the wells to solubilize the dye. The plates were shaken vigorously for 5 min. The absorbance was measured using micro plate reader at a wavelength of 540 nm. The percentage growth inhibition was calculated using the formula below.

$$\%\;\mathrm{Growth}\;\mathrm{inhibition}=\frac{\mathrm{Mean}\;\mathrm{OD}\;\mathrm{of}\;\mathrm{individual}\;\mathrm{test}\;\mathrm{group}\;\times \;100}{\mathrm{Mean}\;\mathrm{OD}\;\mathrm{of}\;\mathrm{control}\;\mathrm{group}}$$

## Abbreviations

GLA: Gamma linolenic acid; FAME: Fatty acid methyl esters.

## Competing interests

The authors declare that they have no competing interests.

## Authors’ contributions

SJ carried out all the studies, analyzed the data and drafted the manuscript. PD and NM carried out the Isolation part. RK and MA helped with the discussion of the data and the correction of the manuscript. KE participated in the study design and helped to draft the manuscript. All authors have read and approved the final manuscript.
